# Characterization and Modeling of Thermal Protective and Thermo-Physiological Comfort Performance of Polymeric Textile Materials—A Review

**DOI:** 10.3390/ma14092397

**Published:** 2021-05-05

**Authors:** Sumit Mandal, Nur-Us-Shafa Mazumder, Robert J. Agnew, Guowen Song, Rui Li

**Affiliations:** 1 Department of Design, Housing and Merchandising, Oklahoma State University, Stillwater, OK 74078-5061, USA; shafa.mazumder@okstate.edu; 2 Fire Protection and Safety Engineering Technology Program, Oklahoma State University, Stillwater, OK 74078-5061, USA; rob.agnew@okstate.edu; 3 Department of Apparel, Events, and Hospitality Management, Iowa State University, Ames, IA 50011-2100, USA; gwsong@iastate.edu (G.S.); ruili@iastate.edu (R.L.)

**Keywords:** polymeric textiles, textile fabrics, thermal protective performance, thermo-physiological comfort performance, hazardous environment, ambient environment

## Abstract

In 2017, more than 60,000 firefighters and oilfield-workers injuries and fatalities occurred while they were working under various thermal hazards such as flame, radiant heat, steam, etc., or due to their significant heat stress related discomfort. The majority of these burn injuries and fatalities results from an inadequate protection and comfort provided by firefighters’ and oilfield-workers’ fire protective polymeric textile materials used in their workwear. Hence, both the thermal protective and thermo-physiological comfort performance of fabrics used in workwear significantly contribute to limit firefighters’ and oilfield-workers’ skin burns and heat stress. Considering this, previous studies have focused on characterizing and developing empirical models to predict the protective and comfort performance based on physical properties of the fabrics. However, there are still some technical knowledge gaps in the existing literature related to this. This paper critically reviewed the literature on characterization and modeling of thermal protective and thermo-physiological comfort performance of fire protective textile fabric materials. The key issues in this field have been indicated in order to provide direction for the future research and advance this scientific field for better protection and comfort of the firefighters and oilfield-workers.

## 1. Introduction

In 2017, the National Fire Protection Association (NFPA) reported 64 firefighter fatalities and 58,835 firefighter injuries in more than 1 million fire incidents occurred in USA [1,2,3]. According to the U.S. Department of Labor statistics, 1566 workers also died from injuries while they were drilling in the oil-and-gas industry and related fields from 2008 through 2017 [4]. In particular, Oklahoma is one of the 10 most wildfire prone states of USA (as per 2018 statistics from the Insurance Information Institute, USA) and the wild- and structural-fires together causes numerous skin burns and heat stress related injuries to our firefighters [5,6]. Additionally, a recent explosion in one of the Oklahoma’s oil-and-gas rigs resulted in deaths of five workers [7]. Notably, the majority of these fatalities and burn injuries results from an inadequate protection and comfort provided by firefighters’ and oilfield-workers’ fire protective polymeric textile materials used in their workwear [8,9].

The thermal protective performance of fire protective textiles is strongly associated with the thermal environments faced by on-duty firefighters and oilfield-workers [10]. In order to understand the performance of fire protective textiles, many researchers have investigated the thermal environments faced by these workers [8,10,11,12,13]. Through these investigations, it has been established that firefighters’ and oilfield-workers’ are exposed to flames, radiant heat, hot surface contact, steam, and hot liquids of varying intensities and durations. In these thermal exposures, the performance of workwear varies depending upon the characteristics of the textile fabrics used in the workwear. Thus, to improve firefighters’ and oilfield-workers’ protection, there is a need to study and understand the performance of the textile fabrics used in the workwear under different thermal exposures. Additionally, it is evident that textile fabrics used in the workwear may not properly transfer the metabolic heat and sweat vapor from wearers’ bodies to the ambient environment. As a result, these fabrics could cause significant heat stress and strain on wearers’ bodies. Eventually, there is a need to study and understand the thermo-physiological comfort performance of fabrics used in the workwear.

Based on the above discussion, both the thermal protective and thermo-physiological comfort performance of fabrics used in workwear significantly contribute to limit firefighters’ and oilfield-workers’ skin burns and heat stress [8,14,15]. Considering this, various test methods that have been standardized by ASTM (American Society for Testing Materials) or ISO (International Organization for Standardization) were used to measure the protective and comfort performance of the fabrics under different thermal exposures and ambient environment [16,17,18,19,20,21,22]. However, these tests are fabric destructive in nature, time consuming, and/or expensive to carry out on a regular basis [23,24,25]. As a result, previous studies have focused on characterizing and developing empirical models to predict the protective and comfort performance based on physical properties of the fabrics [26,27,28,29,30,31,32,33,34,35,36,37,38,39,40,41,42,43,44,45,46,47,48,49,50,51,52,53,54,55,56,57,58,59,60,61,62,63]. For this, first, significant fabrics’ properties that affect the protective and comfort performance of fabrics were identified. Next, these key fabrics’ properties were employed in developing empirical Multiple Linear Regression (MLR) and/or Artificial Neural Network (ANN) models for convenient prediction of the performance. In general, it was found that ANN models can more accurately predict the performance than MLR models.

Previous studies extensively characterized and modeled the thermal protective and thermo-physiological comfort performance of fire protective textile fabrics. However, there are still some technical knowledge gaps in the existing literature related to this. Considering this, our present manuscript critically reviewed the literature on characterization and modeling of thermal protective and thermo-physiological comfort performance of fire protective textile fabric materials. Based on this review, the key issues in this field have been identified for the future research.

## 2. Characterization and Modeling of Thermal Protective Performance of Polymeric Textile Materials

Previous researchers studied the thermal protective performance of fabrics used in workwear under single or specific thermal exposures [64]. In these studies, the thermal protective performance of the fabrics was evaluated using the test methods developed by many national and international organizations such as ASTM, International Organization for Standardization (ISO), and NFPA [16,17,18,19,20,21,22,65]. These studies have also characterized the fabrics in order to recognize and explain fabric properties significantly affecting the thermal protective performance. By employing these significant properties, some of these studies have also developed models for predicting the thermal protective performance of fabrics.

### 2.1. Thermal Protective Performance under Flame Exposure

In the late 1970s and early 1980s, [23,24] analyzed single- and double-layered fabrics in the high intensity flame exposures. They found that the thickness and weight of fabrics affected the thermal protective performance, and that the protection of double-layered fabrics was much higher than that of single-layered fabrics. Barker and Lee (1987) [66], and Shalev and Barker (1983) [67] demonstrated that the thermal protective performance of single-layered fabrics was affected by changes in the intensity of the flame exposure and also by the thickness and weight of the fabrics. Barker and Lee (1987) [66] further explained that the fabric’s density (mass per unit volume) does have a significant impact on thermal protective performance. Here, if the density of a fabric gradually increases, the thermal protective performance proportionately decreases. However, over the density of ~60 kg/m^3^, the thermal protective performance drops very rapidly. This is because, beyond this density, the dead air trapped inside the fabric structure starts conducting the thermal energy toward the wearer’s skin. This situation rapidly lowers the thermal protective performance of the fabric. Furthermore, Morris (1953) [68] explained that when two fabrics are of equal thickness, the one with lower density shows greater thermal protective performance. In this context, it is necessary to remember that the structural properties of two fabrics with the same density can be quite different. One fabric might be loosely woven from tightly twisted, hard yarns and the other might be closely woven from loosely twisted, soft yarns. This variation in structural properties may affect the thermal protective performance of the fabrics. Contextually, Torvi and Dale (1998) [69], and Torvi, Dale, and Faulkner (1999) [70] found that a fabric with high thermal conductivity and low specific heat could quickly transfer thermal energy through it and lower the thermal protective performance. They also noted that such a fabric could decompose in a flame exposure. Here, the thermal decomposition reactions of the fabric are generally endothermic because little oxygen is available for exothermic oxidation reactions to happen [71]. This endothermic decomposition reaction could generate considerable thermal energy depending upon the intensity and duration of the flame exposure. This thermal energy generated by decomposition could also lower the thermal protective performance of the fabric.

**Figure 1:**
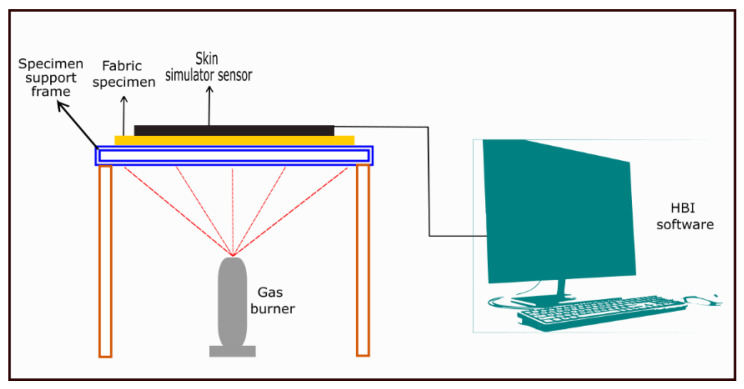
Thermal protective performance evaluating tester under flame exposure.

Mandal et al. (2013) [47] also investigated the thermal protective performance under flame exposure in consideration with fabric properties using modified ISO 9151 test method. The modification was primarily associated with the type of sensor and data calculation technique to predict the thermal protective performance in terms of time required for a second-degree burn injury. In the original ISO 9151 standard, a horizontally oriented specimen of the fabric (14 × 14 cm^2^) is subjected to an incident heat flux of 80 kW/m^2^ from the flame of a gas burner placed beneath it [22]. The heat passing through the specimen is measured by means of a small copper calorimeter placed on top of and in contact with the specimen. The time, in seconds, required to raise the temperature at 24 ± 0.2 °C in the calorimeter is recorded; the mean result for three test specimens is calculated as the ”heat transfer index (flame)”. In the modified ISO 9151 standard, the flame was delivered from a Meker propane gas burner with a diameter of 38 mm (Figure 1) [47]. The burner was adjusted to deliver a heat flux of 84 kW/m^2^. The fabric specimen of size 10 × 10 cm^2^ (Figure 1) was mounted above the burner using the specimen support frame (Figure 1) with the outer layer of the fabric facing the burner. The fabric specimen was protected from the heat source before and after the test run. At the time of the test, the burner was placed beneath the fabric specimen and the flame was delivered for a time that depended on the structure (i.e., the composition and number of layers) of fabric. The thermal energy transferred through the fabric specimen was processed using a skin simulant sensor (Figure 1) mounted on an insulating board and located behind the fabric specimen. The surface (epidermis skin) temperature of the sensor was recorded and the second-degree burn time was calculated using the customized software (Figure 1) that was programmed according to HBI equation. Interestingly, He et al. (2015) [72] mentioned that existing testers cannot properly evaluate the thermal protective performance especially for an exposure to fixed duration. So, they developed an attenuation factor to properly evaluate the thermal protective performance of fabrics.

In this study of Mandal et al. (2013) [47], it was found that a jet of hot gaseous molecules move towards the fabrics surface during the flame exposure; therefore, convection is the primary mode of heat transfer through the fabrics. Authors found that different fabric properties are responsible to transfer the thermal energy through the fabrics and that lower the thermal protective performance of fabrics. In a recent study, Mandal et al. (2018) [41] found that thermal resistance and evaporative resistance are the key fabric properties to affect the thermal protective performance of fabrics under flame exposure. By employing these properties, authors also developed various empirical models such as MLR and ANN models to predict the performance. In this study, it has been found that ANN models could more effectively predict the thermal protective performance of fabrics under flame exposure [46]. Although authors concluded that thermal resistance and evaporative resistance are two important fabric properties that can be used to predict the thermal protective performance under flame exposure using ANN model, authors selected limited number of fabric samples to reach in this conclusion. In the future, a wider range of fabric samples could be selected to characterize and model the thermal protective performance of fabrics. In the future, thermal protective performance could be evaluated in consideration with the attenuation factors and empirical models could be developed for properly predicting the thermal protective performance of fabrics. Moreover, Wang and Li (2015) [73] found that repeated flame exposure to fabric samples continuously reduce the thermal protective performance of the fabrics depending upon the polymeric fibers used in the fabrics. This thermal protective performance also was significantly affected by the shrinkage of the fabrics under flame exposure [74]. In the future, it is necessary to characterize the thermal protective performance of fabrics under repeated flame exposure in consideration with shrinkage to identify the key fabric properties affecting the performance; by utilizing these key properties, empirical models could be developed for predicting the thermal protective performance of fabrics. Recently, Su et al. (2019) [75] studied the thermal protective performance of fabrics using the modified ASTM F 2700 tester. They concluded that depending upon the fabric properties and applied compression on the fabrics, the amount of thermal energy transfer through the fabrics to the wearers’ skins could vary and that can lower the thermal protective performance of the fabrics. However, to date, no modeling approach has been used to evaluate the thermal protective performance of the fabrics in consideration with the applied pressure on the fabrics. Additionally, ASTM F 2700 tester mainly evaluate the thermal protective performance in consideration with two 45° burners and a parallel radiant heat panel relative to the horizontal test fabrics. However, this planar configuration may not be realistic to evaluate the performance. Considering this, Su et al. (2019) [75] developed an equipment to evaluate the performance of fabrics in cylindrical configuration instead of horizontal configuration. It has been found that thermal protective performance of the fabrics could be different in two different configurations of the tested fabrics; however, to date, no modeling approach has been applied to predict the thermal protective performance of fabrics in cylindrical configuration.

In Table 1, a summary of the findings from previous research on thermal protective performance of fabrics under flame exposure is systematically presented.

### 2.2. Thermal Protective Performance under Radiant Heat Exposure

In a bench-top configuration that simulated a combined exposure of flame and radiant heat, Shalev and Barker (1984) [14] observed that the thermal energy transfer rate was lower for thick fabrics than for thin fabrics, and that the air permeability of the fabrics did not significantly affect the transfer of thermal energy. They concluded that air permeability has little or no impact on thermal protective performance of fabrics. Perkins (1979) [76] concluded that fabric weight and thickness are the main properties to consider when analyzing fabric performance in low intensity (~<20 kW/m^2^), radiant heat exposures. Through statistical analysis, he confirmed that fabric weight and thickness are positively associated with the thermal protective performance of fabrics. Fabrics with high thickness entrap more dead air than thinner fabrics, and this air helps to insulate wearers [77,78,79]. However, Song, et al. (2011) [80] observed that thick fabrics store more thermal energy than thin fabrics in the low intensity radiant heat exposures, and this stored energy may be released due to compression during and after the exposure. The release of the stored energy causes burn injury on a wearer’s skin and consequently lowers the performance of the workwear [81,82]. Barker, Guerth-Schacher, Grimes, and Hamouda (2006) [81] stated that fabrics may absorb moisture due to perspiration from a sweating firefighter; thus increasing the thermal conductivity of fibers, and lowering the thermal protective performance of the fabrics [81,83,84]. In contrast, it was also found that if a fabric absorbs a significantly high amount of water (over 15% of its weight), this situation provides a cooling effect to firefighters by reducing the thermal energy transfer [80].

**Figure 2:**
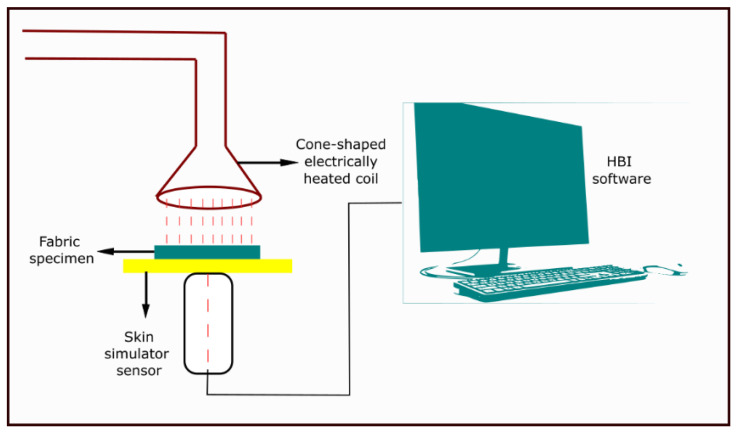
Thermal protective performance evaluating tester under radiant heat exposure.

Mandal et al. (2013) [47] also investigated the thermal protective performance under radiant-heat exposure in consideration with fabric properties using modified ASTM E 1354 test method. The modification involved the use of a data acquisition technique for predicting the time required for a second-degree burn injury as the means of evaluating the thermal protective performance of fabrics. In the original ASTM E 1354 standard, a horizontally oriented specimen of the fabric (10 × 10 cm) is subjected to an incident radiant heat flux of 0–100 kW/m^2^ generated from an electric spark placed on top of it; the ignitability, heat release rates, mass loss rates, effective heat of combustion, and visible smoke development of the specimen in the certain duration exposure are measured using an oxygen consumption calorimeter [65]. In the modified ASTM E 1354 test, heat was generated by a truncated cone-shaped electrically heated (5000 W, 240 V) coil (Figure 2) adjusted to deliver a heat flux of 84 kW/m^2^ [47]. The specimen of the fabrics (15 × 15 cm^2^) (Figure 2) was horizontally mounted beneath the heated coil. The heat flux was kept uniform within the central 50 by 50 mm area of the specimen. A transverse shutter was used to protect the fabric specimen from the heat source before and after the test. The radiant heat exposure time for different fabric specimens was varied according to the structure of the fabric. A skin simulant sensor attached on a frame (Figure 2) was placed behind the test specimen to process the thermal energy transferred through the fabric during the exposure. The surface (epidermis skin) temperature of the sensor was recorded, and the second-degree skin burn time was calculated using the customized and programmed HBI software (Figure 2).

By using the modified ASTM E 1354 test, Mandal et al. (2013) [47] found that fabric thickness is an important property to affect the thermal protective performance of fabrics under radiant heat exposure. Furthermore, Mandal and Song (2014) [46] investigated the thermal protective performance under radiant heat exposure using the same modified test equipment in consideration with a wide range of fabric properties. They found that thickness and thermal resistance of the fabrics significantly affect the protective performance. By using these fabric properties, they also developed the empirical models for predicting the performance. Recently, Mandal et al. (2019) [39] investigated the thermal protective performance (in terms of time to second-degree skin burn injury) of wide range of fabrics under radiant heat exposures of different intensities 10, 40, and 80 kW/m^2^ using ISO 6942 [20] standard test method. They identified that fabric weight is the most significant property to affect the performance in single-layered fabrics whereas thermal resistance is the most significant property to affect the performance of multi-layered fabrics. By using these significant properties, they also developed the empirical models separately for predicting the thermal protective performance of single- and multi-layered fabrics. Onofrei et al. (2014) [85] also developed the mathematical model for heat transfer through the multilayer fabrics used in workwear under low level radiant heat exposure using finite element method. In this model, authors coupled heat transfer through multilayered fabrics with the heat transfer through human skin in order to predict the time to second- and third-degree burn injury on wearers’ bodies. These models also validated the experimental results obtained from ISO 6942 [20] standard. It was found that the models developed by Onofrei et al. (2014) [85] can be successfully used to develop the model for predicting the thermal protective performance of fabrics. In the same direction, Su et al. (2016) [86] modeled the thermal protective performance of multilayered fabrics using finite difference modeling approach. In this study, authors not only considered the transmitted thermal energy through the fabrics under low level radiant heat exposure. They also considered the stored energy within the fabrics under radiant heat exposure in order to develop and validate the model. As this model considered both impact of transmitted and stored thermal energy on the second-degree burn injury, this model can be used for predicting the thermal protective performance of the fabrics. Recently, Puszkarz, Machnowski, and Blasinska (2020) [87] also developed and validated the Computational Fluid Dynamics model for predicting the thermal protective performance of fabrics under radiant heat exposures. However, this model used several assumptions such as the homogenous distribution of solid and air phase in porous fabrics as well as the limited mutual contact within each layer of a multilayered fabrics. These assumptions may not be realistic in real experimental situations of predicting the thermal protective performance of fabrics.

In Table 2, a summary of the findings from previous research on thermal protective performance of fabrics under radiant heat exposure is systematically presented.

### 2.3. Thermal Protective Performance under Hot Surface Contact Exposure

Rossi and Zimmerli (1994) [88] investigated the impact of moisture on thermal protective performance of multi-layered fabrics during hot surface contact. They found that the presence of water in the outer layer of the fabric (exposed to the hot surface contact) enhanced the thermal conductivity of the fabric. As a result, the thermal protective performance of the fabric dropped by 50–60%. In this context, a multi-layered fabric with a separate moisture barrier in the inner layer exhibited better thermal protective performance than a multi-layered fabric with a laminated moisture barrier on the outer shell fabric. However, both of these fabrics exhibited a similar drop in performance when their inner layers were wet. If the inner layer of the fabric was wet, the thermal protective performance was found to drop by 10–25% for all of the selected fabrics of this study. Here, the decrease in thermal protective performance was greater at lower temperatures because the water accumulated in the fabric layers without any significant evaporation, enhancing thermal conductivity and lowering the thermal protective performance of the fabrics. Su et al. (2020) recently concluded that moisture present within the fabrics could significantly affect the performance; however, compression of the fabrics in hot surface contact may not have significant impact of thermal protective performance depending upon the pressure of the compression.

**Figure 3:**
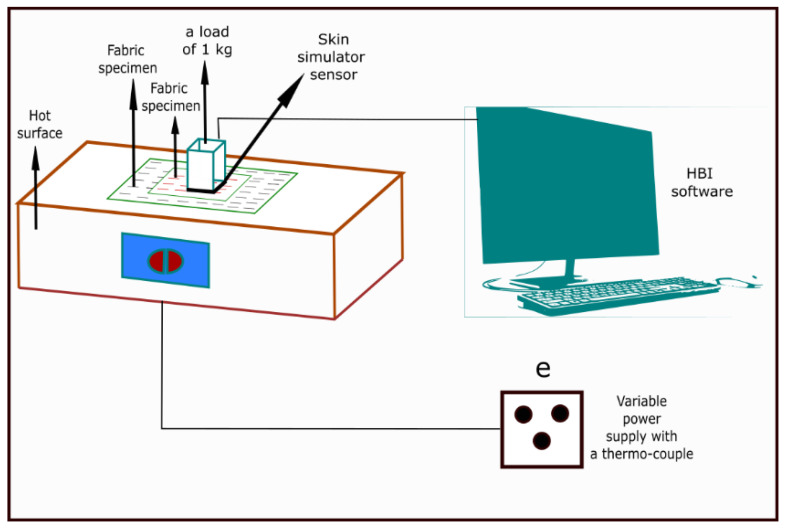
Thermal protective performance evaluating tester under hot surface contact exposure.

Mandal et al. (2013) [47] also investigated thermal protective performance of fabrics under hot surface contact exposure. Thermal protective performance of fabrics in hot surface contact exposure was measured according to a modified ASTM F 1060 (Figure 3) method. The modification was primarily associated with the hot surface temperature, type of sensor, and data calculation procedure to predict the thermal protective performance. In the original ASTM F 1060 standard, a specimen of the fabric system (10 × 15 cm^2^) is horizontally placed in contact (contact-pressure is 3 kPa) with a standard hot surface (temperature is up to 316 °C) [89]. The amount of heat transmitted through the specimen is measured by a copper calorimeter placed on top of the specimen; this calorimeter is mounted in an insulating block with added weight. Finally, the heat measured is compared with the human tissue tolerance (pain sensation or a second-degree burn) and the obvious effects of heat on the specimen (physical damage and degradation) are noted. In the modified ASTM F 1060 test used, the specimen of the fabric system (10 × 15 cm^2^) was placed horizontally (Figure 3) on a hot surface plate of electrolytic copper (Figure 3) under a load of 1 kg (Figure 3) [26]. The temperature of the hot surface (Figure 3) was controlled at 400 °C using variable power supply with a thermocouple (Figure 3). Heat transmitted through the test specimen was processed by a skin simulant sensor (Figure 3) mounted above the fabric specimen on an insulated board. The exposure time varied depending on the composition and number of layers of the fabric system, since the test ran until the transferred energy was sufficient to generate a second-degree skin burn injury. The skin simulant sensor (Figure 3) and customized HBI software (Figure 3) were used to calculate the time required for a second-degree skin burn injury.

Based on the study of Mandal et al., 2013 [47], it was found that thickness is the most important fabric property because fabric with high thickness can trap a lot of dead air and that can provide the insulation under hot surface contact exposure. In the same direction, through a detailed investigation it was also found that thickness and thermal resistance both could be important properties for affecting the thermal protective performance and these properties can be effectively used in ANN modelling techniques for predicting the thermal protective performance. Recently, Mandal and Song (2018) [40] also scientifically developed the theoretical models for explaining the heat transfer through the fabric systems under hot surface contact exposure. They explained that heat mainly transfers from hot surface to the fabrics, within the fabrics, and finally from the fabrics to the wearers’ skin; they provided theoretical models for this heat transfer considering the thermal conductivity and heat capacity of the fabrics. They concluded that thermal protective performance of fabrics differs depending upon the conductive heat transfer mechanism through the fabric system. Although a recent study investigated the thermal protective performance under hot surface contact, there is a still requirement to investigate the performance with a wide range of fabrics and hot surface contact temperatures. This will help to holistically understand the impact of fabric properties and temperatures on the performance under hot surface contact exposure. Recently, Su et al. (2020) [90] also studied the thermal protective performance of fabrics in consideration with the moisture under hot surface contact exposure. In this study, they mentioned that depending upon the moisture content, the heat transferred through the fabrics differs. This is because moisture present in the fabric could significantly store the thermal energy and lower the transmission of the thermal energy towards wearers’ bodies or sensor. As a result, thermal protective performance of the fabrics could increase.

In Table 3, a summary of the findings from previous research on thermal protective performance of fabrics under hot surface contact exposure is systematically presented.

### 2.4. Thermal Protective Performance under Steam Exposure

If moisture that has accumulated inside the fabric structure turns into steam during a thermal exposure, the steam may diffuse toward the skin depending upon the fabric’s characteristics, leading to skin burns [91,92,93,94]. Similarly, water used by firefighters to extinguish fire may generate steam in the environment, and thus, be transferred through their workwear to produce skin burns. Rossi et al. (2004) [94] concluded that water vapor permeability is the most important fabric property to consider for effective protection in steam exposures. They suggested that a water vapor impermeable membrane inside the fabric layers might significantly prevent steam transfer and reduce burn injuries. It was also confirmed that a thick fabric with a water vapor impermeable membrane provides better protection from steam than a thick fabric with a semi-permeable membrane [92,93,95]. Recently, Su et al. (2019) [75] also investigated the impact of different types of membranes on the thermal protective performance of the fabrics under steam exposure. It has been found that thickness, mass and moisture regain of the membranes have significant impact on the performance. Depending upon the surface morphology, water repellency, air permeability and water vapor permeability of the membranes, steam absorption and condensation occurs within the membranes and that lowers the thermal protective performance of the workwear.

**Figure 4:**
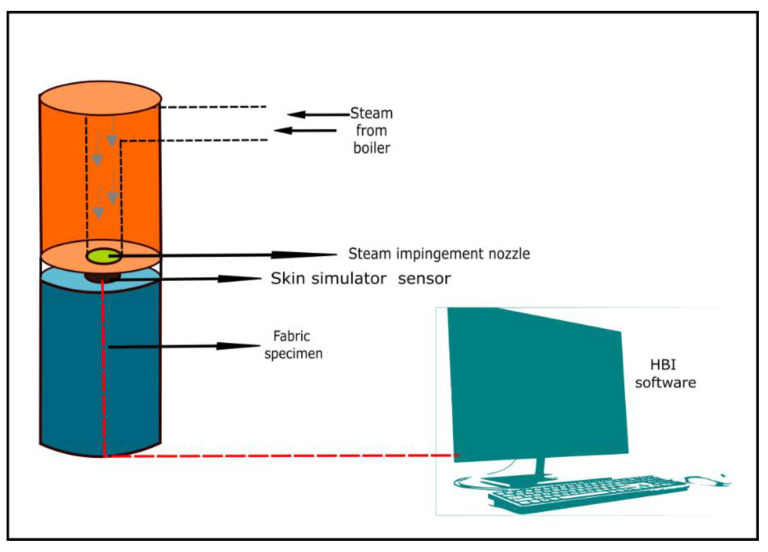
Thermal protective performance evaluating tester under steam exposure.

Mandal et al. (2013) [47] studied the thermal protective performance of fabrics under steam exposure using the tester developed by the research team of Protective Clothing and Equipment Research Facility (PCERF) at the University of Alberta (U of A), Edmonton, Alberta, Canada. A schematic diagram of the steam tester developed by the research team of PCERF) at the U of A is illustrated in Figure 4 [96,97]. Steam (Figure 4) was generated through a 3 kW boiler at a temperature of 150 °C. The fabric specimen (20 × 20 cm^2^) was placed on Teflon plated specimen holder (Figure 4) attached with an embedded skin simulant sensor (Figure 4). The steam was impinged at a pressure of 200 kPa from 50 mm above the fabric specimen through a nozzle having a diameter of 4.6 mm (Figure 4). The duration of the steam exposure was controlled according to the structure of the fabric specimen or system to generate a second-degree burn injury. Notably, although the normal steam exposure time for this tester is 10 s, the steam exposure time was 30 s for the thickest fabric specimen used in this study. During and after the steam exposure, the heat flux through the fabric specimen was processed by the skin simulant sensor and the time required to generate a second-degree skin burn was calculated by the customized and programmed HBI software (Figure 4).

Mandal et al. (2013) [47] and Mandal et al. (2014) [46] found that thickness and air permeability are the two most important properties that can affect the thermal protective performance of fabrics under steam exposure. Nevertheless, air permeability is the most significant property to affect the performance [98]. This is because a fabric with high air permeability could transfer more steam through the porous surface of the fabrics and that could lower the performance of the fabrics. In a recent study, Mandal et al. (2021) [99] investigated the impact of wide range of fabric properties on the performance under steam exposure. It has been found that thickness, air permeability and evaporative resistance of the fabrics are three most significant properties to affect the performance. By employing these properties, Mandal et al. (2021) [99] also developed the MLR and ANN model to predict the performance. It has been found that ANN modelling approach could effectively represents the relationship between these fabric properties and performance under steam exposure. Although this study concluded that ANN modelling approach is useful to predict the performance, this study used limited number and properties of the fabrics. In the future, it is required to extend this study with wide range of fabrics in order to develop an effective model for predicting the performance. By using the similar equipment shown in Figure 4, He, Yu, and Jie (2019) [100] quantified the stored energy within the moistened fabrics under steam exposures. They concluded that fabrics get wet internally and externally under steam exposure and that can store heat; as a result, transmission of the heat gets lower and that can enhance the thermal protective performance of fabrics. However, this study did not develop any model for predicting the thermal protective performance of fabrics in consideration with the moisture.

In Table 4, a summary of the findings from previous research on thermal protective performance of fabrics under steam exposure is systematically presented.

### 2.5. Thermal Protective Performance under Hot Water Exposure

Lu, Song, Ackerman, Paskaluk, and Li (2013) [101], and Lu, Song, Li, and Paskaluk (2013) [102] studied the performance of single-layered fabric systems against hot liquid splash at 85 °C. They used water, drilling mud (manufactured by SAGDRIL), and canola oil to simulate various workplace hazards. They observed that the properties of water, e.g., density, thermal conductivity, surface tension, and heat capacity, at 85 °C were the highest among all liquids evaluated, whereas the dynamic viscosity of water was the lowest of all the liquids at this temperature. They found that the thermal protective performance of the fabric systems evaluated depended on the properties of the fabrics (e.g., weight, thickness, air permeability, fiber content, weave structure) and liquids. They found that the air permeability of a fabric system was negatively associated with thermal protective performance under all types of hot liquid splashes. Although previous studies found the relationship between fabric properties and thermal protective performance under flame and radiant heat exposures, the finding between air permeability and thermal protective performance under hot liquid splash was very limited until 2013 [14,76,101,102]. Lu, et al. (2013) also found that fabric performance was lower when exposed to water or drilling mud than when exposed to canola oil. This was thought to be because the heat capacity of hot-water or drilling mud is higher than the heat capacity of canola oil. Basically, the amount of heat energy per unit mass of hot-water or drilling mud was higher due to their high heat capacity; this high heat content lowered the thermal protective performance of selected fabrics in Lu, et al.’s study [101]. Gholamreza and Song (2013) [103] found that a multi-layered fabric system with an air-impermeable outer layer provided better protection against hot liquid splash than a multi-layered fabric system with an air-permeable outer layer. Few years back, Lu, et al. (2014) [104] investigated the thermal protective performance of different single-layered fabrics under hot liquid splash. They found that the flow pattern of liquids on the fabrics varied depending on the surface energy between the liquid molecule and fabric. Generally, a very hot liquid or highly rough fabric surface could influence the surface tension of the liquid; in turn, increasing the wettability of the fabric. In the case of a fabric with high wettability, the liquid could penetrate through the fabric due to wicking and cause burns on wearers’ skins. Lu, et al. (2014) [104] further mentioned that the liquid applied can be stored in fabric or transmitted through the fabric depending upon fabric properties (thickness, density, air permeability). If a fabric can store more and transmit less liquid, it will show high initial thermal protective performance. They also found that the addition of a thermal liner with a single-layered shell fabric can help to store more and transmit less liquid and this enhances the performance of the shell fabric.

**Figure 5:**
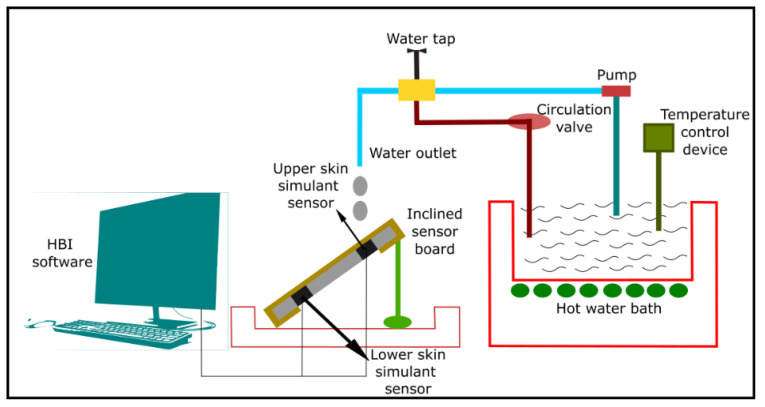
Thermal protective performance evaluating tester under hot water splash exposure.

In some of the previous studies, the hot-water splash test was conducted using a modified ASTM F 2701 (Figure 5) method [42,102,105]. In the original ASTM F 2701 standard, hot-water is hand-poured on the fabric specimen through a funnel to create a 10 s hot-water splash exposure for evaluating the thermal protective performance of the specimen using copper calorimeters [106]. However, Jalbani, et al. (2012) [105] found that this pouring procedure is unrealistic and can affect the hot-water flow rate and repeatability, resulting in an increase in measurement errors. They replaced the funnel with a small pipe, directly fed by a circulating hot-water bath via a small pump through a hose and valve system; this modification provides a consistent application of a given quantity of water at a consistent temperature and flow rate. The equipment was further modified as described by Mandal, et al. (2013) [47] to replace the copper calorimeters with skin simulant sensors. Each fabric specimen (30 × 30 cm^2^) was mounted on an inclined (45°) sensor board (Figure 5) made of a nonconductive, liquid and heat resistant material. The sensor board had two skin simulant sensors—an upper sensor (Figure 5) representing a direct exposure point of the fabric system to the hot-water, and a lower sensor (Figure 5) representing an off-direct exposure point of the fabric system to the hot-water. Notably, only the data obtained from the upper sensor was used for this study. Here, hot-water was prepared in a circulating bath (Figure 5) and its temperature was maintained at 85 °C using a temperature control device (Figure 5). The hot-water was initially circulated by a pump (Figure 5) through a circulation valve attached with a flow control valve (Figure 5) in order to regulate the water temperature within the pipe at 85 °C. Using a water tap (Figure 5), the hot-water was then passed through the water outlet (Figure 5). By employing a thermocouple at the front of the outlet, the water temperature was constantly monitored. Next, the fabric specimen was continuously exposed to the hot-water until a second-degree burn was predicted. The duration of the water flow depended upon the structure of the fabric specimen or system being tested. The thermal energy (in the form of heat and mass transmitted through the specimen) at the direct exposure point was processed using the skin simulant sensor (Figure 5). The surface (epidermis skin) temperature of the sensor was recorded and used to calculate the time required for a second-degree skin burn injury using the customized and programmed HBI software (Figure 5).

Mandal et al. (2013) [47], Mandal et al. (2014) [45] investigated the thermal protective performance of fabrics under hot water splash exposure. It has been found that thickness, air permeability, and/or evaporative resistance are the most significant properties to affect the performance. Mandal (2016) [26] also employed these properties for to develop the models for predicting the performance. It has been concluded that the ANN modeling methodologies could be good fit for predicting the performance; however, there is a need to investigate this in consideration with wide range of single- and multi-layered fabrics for accurately and conveniently predicting the performance.

**Figure 6:**
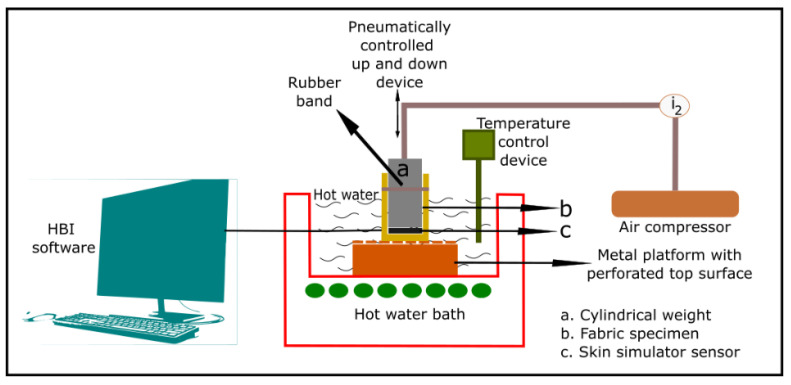
Thermal protective performance evaluating tester under hot water immersion with compression exposure.

Previous researchers focused on the thermal protective performance of fabrics under hot-water splash conditions [101,102,103,104]. However, on-duty firefighters are not so likely to be exposed to hot-water splash only. They do kneel and crawl on the floor while working to extinguish fires and rescue fire-victims. While performing these activities, their workwear is compressed specifically in the knees, elbows, and lower-legs. The workwear may also be immersed in hot-water. This hot-water immersion with compression can cause skin burns to firefighters’ arms, hands, legs, and feet [10,107]. Burn injury statistics indicated that nearly 38% of burn injuries occurred on firefighters’ arms/hands and legs/feet during the period 2007–2011 in the U.S. [108]. Considering this, the hot-water immersion with compression test was carried out using a new test apparatus available at the University of Alberta (U of A), Edmonton, Alberta, Canada in several recent studies [26,42,43]. In these studies, a metal platform with perforated top surface (Figure 6) was positioned at the bottom-center of a hot-water bath (Figure 6). Then, water (Figure 6) was poured into the bath up to a level 6 cm above the perforated top surface. The water temperature was maintained at 75 °C, 85 °C, or 95 °C using a temperature control device (Figure 6). Next, a 30.5 × 30.5 cm^2^ fabric specimen (Figure 6) was attached with a rubber band (Figure 6) to the skin simulant sensor (Figure 6) mounted on a cylindrical weight (Figure 6). This specimen-covered sensor was immersed into the hot-water bath using a pneumatic device (Figure 6) until the whole assembly (specimen + sensor) rested flatly on the center of the perforated surface. Pressure was applied to compress the specimen between the sensor and perforated surface and was pneumatically controlled at 14 kPa (~2.0 psi), 28 kPa (~4.0 psi), or 56 kPa (~8.0 psi). Thermal energy transmitted through the compressed specimen was processed by the sensor for a period of 120 s. From the thermal energy, time required to generate a second-degree skin burn was calculated by the customized HBI software (Figure 6). In the recent study, Mandal et al. (2021) [99] concluded that thickness, air permeability and evaporative resistance are the most important properties to affect the performance of fabrics under hot water immersion and compression exposures of different temperatures and pressures. By employing these properties, it is also possible to predict the performance of the fabrics. However, there is a need to evaluate the performance of wide range of fabrics under different temperatures and pressures exposed by wearers in this hot water immersion and compression condition.

In Table 5, a summary of the findings from previous research on thermal protective performance of fabrics under hot water exposure is systematically presented.

## 3. Characterization and Modeling of Thermo-Physiological Comfort Performance of Polymeric Textile Materials

**Figure 7:**
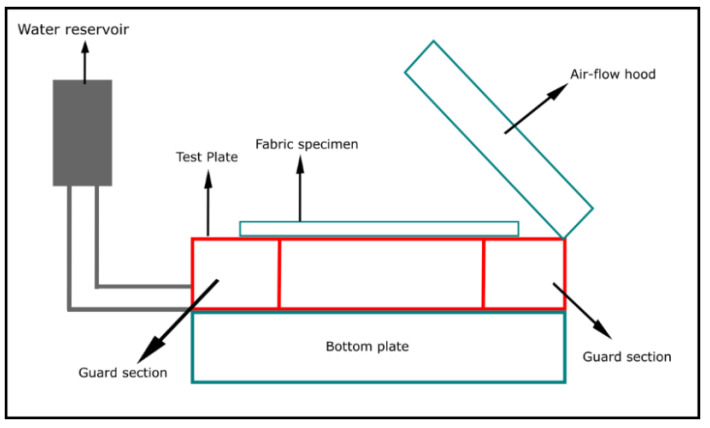
Thermo-physiological comfort performance evaluating tester—Sweating Guarded Hot Plate.

Thermo-physiological comfort performance of fire protective textile materials can be evaluated by using Sweating Guarded Hot Plate (SGHP) method as per the ASTM F 1868 standard (Figure 7). In this standard, thermal resistance (Rct), evaporative resistance (Ret), and Total Heat Loss (THL) are measured in order to evaluate the thermo-physiological comfort performance of fabrics. A detail of this method has been described by Song, Mandal, and Rossi (2016) [27].

There are several limitations of the ASTM F 1868 method. For example, in order to evaluate R_ct_ and R_et_, it is necessary to reach steady-state conditions for the tested fabrics and that could be difficult for some fabrics; in the presence of the 1 m/s ambient air velocity during the testing, tested fabric samples could lift off from the plate and that can result in abnormally high R_ct_, high R_et_ and low THL; contextually, this standard does not indicate the direction and level of air flow or turbulence and it is a very cumbersome process to calculate the THL. Furthermore, ASTM F 1868 standard measures the R_ct_ and R_et_ individually; however, it is necessary to evaluate the R_ct_ and R_et_ together as metabolic heat and sweat vapor transfers from wearers’ bodies occur simultaneously in real situations [109]. Additionally, SGHP equipment did not consider the physiological state of the wearer and are inadequate to evaluate the transient thermal properties of workwear. Keeping this in mind, Psikuta et al. (2013) [110] have developed a thermo-physiological human simulator sweating torso that can realistically measure the thermos-physiological comfort performance of fabrics used in workwear. By using this human simulator, ISO currently introduced two new standards for evaluating the thermo-physiological comfort performance of fabrics—(1) ISO 18640-1:2018 Protective clothing for firefighters—Physiological impact—Part 1: Measurement of coupled heat and moisture transfer with the sweating torso; and, (2) ISO 18640-2:2018 Protective clothing for firefighters—Physiological impact—Part 2: Determination of physiological heat load caused by protective clothing worn by firefighters.

One of the main requirements of fire protective textile fabrics used in workwear is high R_ct_ in order to provide protection to wearers in thermal exposures. Nevertheless, it is necessary to maintain a balanced R_ct_ for better protection and comfort of wearers [111,112,113]. It is evident that the weaving structure of a fabric could significantly affect the thermal resistance of the when the densities of warp and weft yarns are same—for example, R_ct_ of plain weave fabrics are lower in comparison to the rep, twill, or hopsack fabric; however, R_ct_ of the fabrics can be varied only by changing the linear density of the weft yarn [114,115]. Gibson (1993) [116] studied the R_et_ of various permeable and impermeable woven and nonwoven fabrics with single-layered, laminates, and composites structures in consideration with air velocity, air flow direction and air gap exist between the samples and hot plate. It has been found that impermeable materials restrict the transferring of moisture vapor to the preamble fabric structures; eventually, R_et_ is significantly high for these materials. The opposite phenomenon has been observed in the case of permeable fabric due to the easy transfer of the moisture through the fabric structure and air flow/gaps play significant role on R_et_ in permeable fabric. Havenith, Hartog, and Martini (2011) [117] found that the membrane present in any multilayered fabrics could significantly restrict the moisture transfer from wearers bodies to their environment and that can significantly increase the R_et_ of the workwear. This situation causes significant heat stress and strain to wearers’ bodies. Recently, Tian, et al. (2012) [118] investigated the THL through the multilayered fabrics in consideration with the R_ct_ and R_et_. It has been found that in the case of multilayered fabrics, the layer that is in contact with the hot plate plays an important role to the heat loss from wearer’s body to the environment. Here, transient heat transfer mainly occurs and the arrangement of the layers in the fabrics are main considering factors to transfer the heat properly depending upon the volumetric heat capacity of fabrics.

Recently, Guan et al. (2019) [37] studied the sweat transfer through the fabrics in consideration with the material properties, external radiant heat and internal metabolic heat by using the sweating torso. In this study, it has been found that the sweating induces evaporative cooling and increases the external radiant heat transfer to the wearers’ body for hydrophobic materials. On the other hand, the perspired moisture can increase evaporative transfer of sweat moisture and decrease in radiant heat gain for hydrophobic materials. In addition, fabric R_ct_ and R_et_ are important property along with fabric thickness and emissivity when assessing metabolic heat dissipation and radiant heat gain while the wearers are sweating profusely in a working condition. In another study, Guan et al. (2019) [38] indicated that sweat evaporation rate through the fabrics increases for fabrics with high evaporative resistance when high amount of moisture accumulated within a hydrophilic fabric; however, after a certain time, the evaporation rate decreased for the fabric due to the reduction in the mass transfer coefficient of the fabrics.

Recently, Mandal et al. (2019) [61] evaluated the thermo-physiological comfort performance of wide range of fabrics used in workwear. In this study, ISO 18640-1:2018 and ISO 18640-2:2018 standards were used to evaluate the thermo-physiological comfort performance of fabrics in terms of time required to generate heat stress on wearers’ bodies. In this study, it has been found that fabric weight, water spreading speed, and evaporative resistance are the significant properties to affect the thermo-physiological comfort performance of fabrics. By using these properties, Mandal et al. (2019) [61] also developed the MLR and ANN models for predicting the thermo-physiological comfort performance of fabrics; it has been found that ANN model could perform better for predicting the thermo-physiological comfort performance of fabrics.

## 4. Key Issues in the Field of Thermal Protective and Thermo-Physiological Comfort Performance of Polymeric Textile Materials

Previous studies characterized the polymeric fabrics to identify the key fabric properties affecting the thermal protective and thermo-physiological comfort performance of fabrics. By employing these properties, some of these studies develop the models and recommended using ANN models to predict the performance. However, these models were developed based on the experimental thermal protective performance values of dry fabrics only. As workers (firefighters, oilfield-workers) sweat profusely while firefighting, this sweat moisture could affect the thermal protective performance of fabrics [88,119]. Additionally, previous studies were carried out without considering any air gap and resulting microclimates between the fabrics and wearers’ bodies. However, to reproduce more realistic conditions, microclimate air gap must be considered as it can substantially influence the thermal protective and thermo-physiological comfort performance of fabrics [107,120,121]. Contextually, Fu et al. (2015) [122] tried to develop the model to analyze heat and moisture transfer through the fabrics in consideration with moisture and air gap; however, these models cannot be properly used to predict the thermal protective performance of fabrics in terms of time to second- and third-degree burns on wearers’ bodies. Thus, it suggests extending characterization and modeling studies on protective and comfort performance by considering the moistened fabrics and microclimate air gaps. Additionally, previous studies only evaluated the thermal protective performance and thermo-physiological under limited thermal exposures intensity and ambient environment. In the future, it is necessary to study the thermal protective and thermo-physiological comfort performance of fabrics under wide range of thermal exposures, intensities, and ambient environment in consideration with the moisture and air gaps. This could help to holistically understand the thermal protective and thermo-physiological comfort performance of fabrics. Furthermore, previous studies mainly focused on empirical models to predict the thermal protective and thermo-physiological comfort performance of fabrics. Only limited studies have focused on other modeling approaches such as Computational Fluid Dynamics, Finite Element Method, etc., [85,86]. In the future, it is necessary to develop the model using state-of-the-art modeling approach for predicting the thermal protective and thermo-physiological comfort performance of fabrics.

Furthermore, extensive research has investigated the thermal protective and thermo-physiological comfort performance of fabrics used in the workwear under different thermal exposures (e.g., flame, radiant heat) and ambient environments (warm to cold temperature, high to low relative humidity). However, for the certification purpose, thermal protective performance of workwear gets more emphasis instead of thermo-physiological comfort performance by all available product standards. By changing the fabric properties, it is possible to increase the thermal protective performance of fabrics; however, a fabric with high thermal protective performance generally possesses a low thermo-physiological comfort performance. As these performances are inversely related, there is a need for a categorization tool based on both thermal protective and thermo-physiological comfort performance. This kind of tool could help in finding the best balance between these two performances, which could guide workwear manufacture to select an appropriate fabric for the workwear based on their requirements for end uses. Considering this, recently Mandal et al. (2019) [61] conducted a study on categorizing the fabrics based on their thermal protective and thermo-physiological comfort performances. However, in this study, the categorization tool was developed mainly based on the thermal protective and thermo-physiological comfort performances values of dry fabrics without any consideration of the air gaps. In the future, it is recommended to develop the categorization tool based on thermal protective and thermo-physiological comfort performance moistened fabrics and air gaps for more realistic simulations. Based on the optimum protective and comfort performance, it is also possible to develop new polymeric textile materials by using the nanotechnology, aerogel, smart textiles, etc., [123,124]. This new materials-based workwear could provide better protection and comfort to firefighters and oilfield-workers.

## Figures and Tables

**Table 1:** Thermal protective performance under flame exposure.

Author	Findings
Benisek and Philips (1979 & 1981) [23,24]	Thickness and weight of fabrics affected the thermal protective performance; double layered fabrics had much higher than the single-layered fabrics.
Barker and Lee (1987) [66] & Shalev and Barker (1983) [67]	Thermal protective performance of single-layered fabrics was affected by changes in the intensity of the flame exposure, the thickness and weight of the fabric also affected the thermal protective performance.
Morris (1953) [68]	Lower density shows greater thermal protective performance when two fabrics are of equal thickness.
Torvi and Dale (1998) [69], and Torvi, Dale, and Faulkner (1999) [70]	Lower thermal protective performance showed by the fabrics with high thermal conductivity and low specific heat.
Mandal et al. (2018) [41]	Thermal protective performance of fabrics under flame exposure mostly effected by the thermal resistance and evaporative resistance of the fabrics.
Wang and Li (2016) [73]	Repeated flame exposure could reduce the thermal protective performance depending on the types of fibers used in the fabrics.
Wang et al. (2016) [73]	Thermal protective performance significantly affected by the shrinkage of the fabrics.
Su et al. (2019) [75]	The amount of thermal energy transfer through the fabrics depends on the fabric properties and applied compression.

**Table 2:** Thermal protective performance under radiant heat exposure.

Author	Findings
Shalev and Barker (1984) [14]	Thermal energy transfer rate was lower for thick fabrics than for thin fabrics. Air permeability has little or no impact on thermal protective performance of fabrics.
Perkins (1979) [76]	Fabric weight and thickness are positively associated when analyzing fabric performance in low intensity (~<20 kW/m^2^), radiant heat exposures.
Sun et al. (2000), Torvi and Dale (1999), Fanglong et al. (2007) [77,78,79]	Entrapped air within the fabrics helps to insulate wearers.
Song, et al. (2011) [80]	Thick fabrics store more thermal energy, which may be released during compression and cause burn injuries. Significant high amount of absorbed moisture could provide cooling effect by reducing the thermal energy transfer.
Barker et al. (2006) [81]	Moisture from perspiration increase the thermal conductivity, which reduce the thermal protective performance.
Mandal et al. (2013) [47]	Fabric thickness is an important property to affect the thermal protective performance under radiant heat exposure
Mandal and Song (2014) [46]	Thickness and thermal resistance of the fabrics significantly affect the protective performance
Mandal et al. (2019) [39]	Fabric weight is the most significant property to affect the performance in single-layered fabrics. Thermal resistance is the most significant property to affect the performance of multi-layered fabrics.
Onofrei et al. (2014) [85]	The models developed by the authors can be successfully used to develop the model for predicting the thermal protective performance of fabrics.
Su et al. (2016) [86]	Stored energy within the fabrics was also considered in model developing; this model can be used for predicting the thermal protective performance of the fabrics.

**Table 3:** Thermal protective performance under hot surface contact exposure.

Author	Findings
Rossi and Zimmerli (1994) [88]	Presence of water in the outer layer of the fabric increased thermal conductivity in hot surface contact, which decreased the thermal protective performance.
Mandal et al., 2013 [47]	Fabric with high thickness can trap a lot of dead air and that can provide the insulation under hot surface contact exposure.
Mandal and Song (2018) [40]	Developed the theoretical models for explaining the heat transfer through the fabric systems under hot surface contact exposure.
Su et al. (2020) [90]	Moisture present in the fabric could significantly store the thermal energy and lower the transmission of the thermal energy towards wearers’ bodies or sensor.

**Table 4:** Thermal protective performance under steam exposure.

Author	Findings
Rossi et al. (2004) [94]	Water vapor permeability is the most important fabric property while considering protection in steam exposure.
Keiser and Rossi (2008), Keiser et al. (2010), Sati et al. (2008) [92,93,95]	fabric with a water vapor impermeable membrane provides better protection from steam than a fabric with a semi-permeable membrane.
Mandal et al. (2013) [47] and Mandal et al. (2014) [46]	Thickness and air permeability are the two most important property that can affect the thermal protective performance of fabrics under steam exposure.
Mandal et al. (2021) [99]	Thickness, air permeability and evaporative resistance of the fabrics are three most significant properties to affect the performance in steam exposure. MLR and ANN models also have been developed to predict the performance.
He, Yu, and Jie (2019) [100]	Fabrics get wet internally and externally under steam exposure and that can store heat, which lowers the transmission of heat and thereby enhanced the thermal protection.

**Table 5:** Thermal protective performance under hot water exposure.

Author	Findings
Lu et al. (2013) [101] and Lu et al. (2013) [102]	Thermal protective performance of the fabric systems depended on the properties of the fabrics (i.e., weight, thickness, air permeability, fiber content, weave structure) and liquids.
Lu et al. (2013) [101]	Fabric performance was lower when exposed to water or drilling mud than when exposed to canola oil.
Gholamreza and Song (2013) [103]	Multi-layered fabric system with an air-impermeable outer layer provided better protection against hot liquid splash than a multi-layered fabric system with an air-permeable outer layer.
Lu, et al. (2014) [64]	Fabric with high wettability, the liquid could penetrate through the fabric due to wicking and cause burns on wearers’ skins.
Jalbani, et al. (2012) [104]	Found that this pouring procedure in ASTM F 2701 standard is unrealistic and can affect the hot-water flow rate and repeatability. Their modified process provides a consistent application of a given quantity of water at a consistent temperature and flow rate.
Mandal, et al. (2013) [47]	Further modified the equipment introduced by Jalbani, et al. (2012) [104] to replace the copper calorimeters with skin simulant sensors.
Mandal et al. (2013) [47], Mandal et al. (2014) [45]	Found that thickness, air permeability, and/or evaporative resistance are the most significant properties to affect the performance of fabrics under hot water splash exposure.
Mandal (2016) [26]	Employed most significant properties to affect the performance of fabrics under hot water splash exposure to develop the models for predicting the performance. The authors concluded that the ANN modeling methodologies could be good fit for predicting the performance.
Mandal (2016) [26], Mandal et al. (2016) [42], Mandal et al. (2016) [43]	Hot-water immersion with compression test was carried considering the compression specifically in the knees, elbows, and lower-legs during kneel and crawl.
Mandal et al. (2021) [99]	Thickness, air permeability and evaporative resistance are the most important properties to affect the performance of fabrics under hot water immersion and compression exposures of different temperatures and pressures.
